# Comportamento da mortalidade por câncer de mama nos municípios brasileiros e fatores associados

**DOI:** 10.26633/RPSP.2017.168

**Published:** 2017-12-05

**Authors:** Maria Silvia de Azevedo Couto, Maximiliano Ribeiro Guerra, Vinícius de Azevedo Couto Firme, Maria Teresa Bustamante-Teixeira

**Affiliations:** 1 Universidade Federal de Juiz de Fora (UFJF) Programa de Pós-Graduação em Saúde Coletiva Juiz de Fora (MG) Brasil Universidade Federal de Juiz de Fora (UFJF), Programa de Pós-Graduação em Saúde Coletiva, Juiz de Fora (MG), Brasil.; 2 Inserm U900, Institut Curie, PSL Research University Paris França Inserm U900, Institut Curie, PSL Research University, Paris, França.; 3 Universidade Federal de Juiz de Fora – Campus Governador Valadares (UFJF/GV) Governador Valadares (MG) Brasil Universidade Federal de Juiz de Fora – Campus Governador Valadares (UFJF/GV), Governador Valadares (MG), Brasil.

**Keywords:** Neoplasias mamárias, mortalidade, epidemiologia, métodos e procedimentos estatísticos, Brasil, Breast neoplasms, mortality, epidemiology, statistical methods and procedures, Brazil, Neoplasias de la mama, mortalidad, epidemiología, métodos y procedimientos estadísticos, Brasil

## Abstract

**Objetivo.:**

Analisar o comportamento da mortalidade por câncer de mama nos municípios brasileiros e avaliar a influência de fatores socioeconômicos e demográficos sobre as taxas e mortalidade.

**Métodos.:**

Foram calculadas taxas de mortalidade, padronizadas por faixa etária e corrigidas por causas mal definidas, centradas em 1990, 2000 e 2010. Posteriormente, foram estimados modelos de regressão, com dados em painel, que permitiram verificar o grau de associação entre os fatores de interesse e a taxa de mortalidade pela doença.

**Resultados.:**

Verificou-se uma tendência de crescimento da mortalidade no país. Contudo, os modelos indicaram que a mortalidade poderia ter diminuído (tendência negativa), principalmente no Sudeste e Sul, caso alguns fatores associados à doença (por exemplo, nível de renda, educação, longevidade, taxa de fecundidade, gastos em saúde, infraestrutura, entre outros) tivessem permanecido constantes durante o período considerado. Observou-se que a mortalidade por câncer de mama apresentou associação positiva/significativa com a longevidade e negativa/significativa com o nível de gastos públicos em saúde. A mortalidade foi maior nas regiões Sul e Sudeste, nos municípios com mais de 500 000 habitantes e naqueles onde a população é inferior a 5 000.

**Conclusões.:**

O crescimento da renda per capita, a elevação da expectativa de vida e a diminuição da taxa de fecundidade podem estar associados a elevadas taxas de mortalidade por câncer de mama e a uma tendência de crescimento na mortalidade por esse câncer nos municípios brasileiros.

## Abstract (en)

**Objective.:**

To analyze breast cancer mortality trends in Brazilian municipalities and assess the influence of socioeconomic and demographic factors on mortality rates.

**Methods.:**

Age-adjusted mortality rates were calculated for the periods centered in 1990, 2000, and 2010 and corrected for ill-defined causes of death. After that, panel data regression models were developed for analysis of the association between the factors of interest and the mortality rate from breast cancer in Brazilian municipalities

**Results.:**

A growing trend was detected in breast cancer mortality in Brazil. However, the models showed that the mortality could have decreased (negative trend), especially in the Southeast and South regions, if some associated factors (such as income, education, longevity, fertility rate, health spending, and infrastructure, among others) had remained constant during the study period. Breast cancer mortality was positively/significantly associated with longevity and negatively/significantly associated with public health spending. Mortality was higher in the South and Southeast, in municipalities with more than 500 000 inhabitants and in those with population below 5 000.

**Conclusions.:**

The growth in *per capita* income, the increase in life expectancy, and the decrease in fertility rates may be associated with high breast cancer mortality and a trend towards increased mortality from this cancer in Brazilian municipalities.

## RESUMEN

**Objetivo.:**

Analizar las tendencias de la mortalidad por cáncer de mama en municipios brasileños y evaluar la influencia de factores socioeconómicos y demográficos sobre las tasas de mortalidad.

**Métodos.:**

Se calcularon las tasas de mortalidad, ajustadas por grupo etario y corregidas por causas mal definidas, para los años 1990, 2000 y 2010. Posteriormente, se calcularon modelos de regresión, a partir de datos de panel, que permitieron verificar el grado de asociación entre distintos factores de interés y la tasa de mortalidad por esta enfermedad.

**Resultados.:**

Se verificó que había una tendencia al aumento de la mortalidad en el país. Sin embargo, los modelos indicaron que la mortalidad podría haber disminuido (tendencia negativa), principalmente en el sudeste y el sur, si algunos factores asociados (por ejemplo, nivel de ingresos, educación, longevidad, tasa de fecundidad, gastos en salud, infraestructura, entre otros) hubieran permanecido constantes durante el período en estudio. Se observó que la mortalidad por cáncer de mama presentó una asociación positiva y significativa con la longevidad, y negativa y significativa con el nivel del gasto público en salud. La mortalidad fue mayor en las zonas sur y sudeste, en los municipios con más de 500 000 habitantes y en aquellos cuya población es inferior a 5000.

**Conclusiones.:**

El aumento de los ingresos per cápita, el incremento de la esperanza de vida y la disminución de la tasa de fecundidad pueden estar asociados a tasas altas de mortalidad por cáncer de mama y a una tendencia al aumento de dicha mortalidad en los municipios brasileños.

Ao contrário do que ocorre em boa parte dos países desenvolvidos, que apresentam tendência de aumento da incidência e de redução da mortalidade por câncer demama (1-6), no Brasil a taxa de mortalidade por esse tipo de câncer cresceu nas últimas três décadas (7). Estudos indicam que a mortalidade por câncer de mama no país aumentou sistematicamente (8) em diferentes faixas etárias (7, 9, 10). Contudo, esse crescimento não foi homogêneo, e algumas localidades apresentaram, inclusive, uma redução na mortalidade pela doença (7, 10).

A literatura reconhece que a taxa de mortalidade por câncer de mama varia conforme a região e o período analisados (10-12). Essa heterogeneidade regional pode ser um reflexo de características geográficas, demográficas, socioeconômicas e culturais (2, 13-15). No Brasil, uma associação positiva foi relatada entre nível de renda e risco, incidência e mortalidade por câncer de mama (13, 16). Por outro lado, estudos sugerem que maiores níveis de escolaridade poderiam reduzir o risco associado ao câncer de mama (17), aumentar a sobrevida e facilitar o tratamento e detecção precoce da doença, contribuindo para a diminuição damortalidade (18). Ademais, a escassez e a má distribuição de recursos físicos e humanos prejudicam o atendimento e o prognóstico da doença, principalmente em localidades de menor porte e afastadas das capitais. Esse fato pode sobrecarregar os centros oncológicos de referência, concentrados em localidades de grande porte (19, 20). Finalmente, os processos de industrialização também podem contribuir para aumentar o risco associado à doença pela exposição a fatores químicos, físicos e biológicos (11, 21).

Assim, o presente estudo teve por objetivo analisar o crescimento da mortalidade por câncer de mama observado em diferentes localidades do Brasil e verificar a influência dos fatores associados a essa neoplasia.

## MATERIAIS E MÉTODOS

Trata-se de um estudo ecológico que utilizou os municípios brasileiros como unidades de análise e considerou as taxas médias de mortalidade por câncer de mama, padronizadas por idade, centradas nos anos de 1990 (média de 1987 a 1993), 2000 (média de 1997 a 2003) e 2010 (média de 2007 a 2013). Foram utilizados dados secundários não identificados, disponibilizados pelos órgãos responsáveis por sua coleta e divulgação, o que garante a confidencialidade.

### Fonte dos dados

Foram utilizados os óbitos femininos por neoplasia maligna da mama ocorridos nos municípios brasileiros (tendo como referência a residência das falecidas) disponibilizados pelo Sistema de Informação sobre Mortalidade (SIM) do Departamento de Informática do SUS (DATASUS). Esses óbitos foram desagregados pelas faixas etárias da Organização Pan-Americana da Saúde (OPAS). Até 1995, o código 174 da Nona Revisão da Classificação Internacional de Doenças (CID-9) foi utilizado para identificar os óbitos. A partir de 1995, foi utilizado o código C50 da CID-10 (22).

Visando a minimizar o impacto da inexistência de registros de óbito por câncer de mama em alguns municípios, foram utilizadas as médias dos óbitos acumulados de 1987 a 1993 (CID-9), 1997 a 2003 (CID-10) e 2007 a 2013 (CID-10), centradas em 1990, 2000 e 2010, respectivamente. Tais médias foram utilizadas no cálculo da correção por causas mal definidas.

Como os óbitos por causas mal definidas podem comprometer a análisefidedigna das estatísticas de mortalidade (23), utilizou-se um procedimento (24) que consiste em calcular o percentual de correção por causas mal definidas (PCCMD) para cada município “i” no período “t” e o seu respectivo fator de correção, conforme as equações 1 e 2:

$$PCCMD_{it}=\frac{\left({\text{totalobitosfem}}_{it}-{\text{obitoscausasexternas}}_{it}\right)}{\left[\left({\text{totalobitosfem}}_{\text{it}}-{\text{obitoscausasexternas}}_{it}\right)-{\text{obitosmaldefinidos}}_{it}\right]}[1]$$

Após o cálculo do PCCMD, multiplicase o fator de correção de cada município “i”, no período “t” (FC_it_), pelo total de óbitos segundo faixa etária desse mesmo município no período “t”. Assim, obtêm-se os óbitos corrigidos por causas mal definidas, tornando possível o cálculo das taxas específicas de mortalidade por câncer de mama (MCM_it_) por faixa etária e das taxas corrigidas por causas mal definidas. Portanto, as MCM_it_correspondem à média de óbitos corrigidos por causas mal definidas de cada município nos períodos citados, dividida pela população do município calculada pelo Instituto Brasileiro de Geografia e Estatística (IBGE) e disponibilizada pelo Instituto de Pesquisa Econômica Aplicada (IPEADATA) (25).

Para compor os óbitos por causas mal definidas (equações 1 e 2), utilizaram-se os óbitos femininos do capítulo XVI da CID-9 (sintomas, sinais e afecções mal definidas) para o período de 1987 a 1993. Para o período de 1997 a 2013, foram considerados os óbitos femininos do capítulo XVIII da CID-10 (sintomas, sinais e achados anormais de exames clínicos e de laboratório). Os óbitos totais, óbitos por causas mal definidas e óbitos por causas externas são disponibilizados pelo SIM através do DATASUS (22) e foram desagregados por faixa etária da OPAS.

As taxas de mortalidade, corrigidas por causas mal definidas, foram padronizadas por faixa etária via método direto, usando a população padrão mundial, estratificada por faixa etária (26). Considerando as variáveis mencionadas na introdução, determinou-se a equação 3:

$$MCM_{it}=f\left[\begin{array}{c} IDHE_{it},IDHL_{it},IDHR_{it},IND_{it},FEC_{it},MJF_{it},\\ \left(\frac{PR_{it}}{PU_{it}}\right),GP_{it},NES_{it},NPS_{it},DPM_{i},DG_{i},TEND_{t} \end{array}\right]$$

onde MCMit representa a taxa MCM do município “i” no período “t” (sendo t = 1990, 2000 e 2010).

Quanto às demais variáveis da equação 3, o índice de desenvolvimento humano (IDH) varia de 0 (pior cenário) a 1 (melhor cenário) e mede o grau de desenvolvimento socioeconômico de cada município. Os IDH municipais referentes a educação (IDHE), longevidade (IDHL) e renda (IDHR) foram obtidos do Atlas de Desenvolvimento Humano (27), assim como a taxa de fecundidade, o percentual de mulheres jovens (menores de 18 anos) com filhos e a proporção da população rural em relação à urbana.

Para a industrialização, utilizou-se o produto interno bruto (PIB) *per capita* municipal da indústria (valor adicionado a preços básicos), calculado pelo IBGE e convertido em valores constantes de 2000 com base no Índice Nacional de Preços ao Consumidor Amplo (IPCA) do IPEADATA. O gasto público *per capita* em saúde é fornecido pela Secretaria do Tesouro Nacional. Tanto os dados de industrialização quanto o gasto público *per capita* são disponibilizados pelo IPEADATA (25).

O número de estabelecimentos de saúde e de profissionais de saúde com ensino superior em cada município (por 100 000 habitantes) estão disponíveis no TabNet, módulo rede assistencial do DATASUS (22). Para 1991 e para 2000, foram utilizados os dados da Pesquisa Assistência Médico-Sanitária de 1992 e 1999, respectivamente. Os dados de 2010 estão no Cadastro Nacional de Estabelecimentos de Saúde.

Foram criadas *dummies* geográficas para os municípios das regiões Sul, Centro-Oeste, Nordeste e Norte. Por esse procedimento, todos os municípios pertencentes à região em análise recebem valor de 1, enquanto os municípios das outras regiões recebem o valor de zero – de forma que seja possível captar o efeito de pertencer auma região específica, permitindo verificar se a taxa de mortalidade é diferente entre regiões. O Sudeste foi tratado como referência. Também foram criadas *dummies* de porte populacional. Nesse último caso, os municípios “i” foram subdivididos de acordo com porte em i<5; 5≤i<10; 10≤i<20; 20≤i<50; 50≤i<100;100≤i<500; 500≤i<1 000; e i≥1 000, onde os valores representam milhares de habitantes (os municípios com menos de 5 000 habitantes, i<5, foram considerados como referência).

As categorias de referência servem como base de comparação. Assim, a constante estimada refletirá a taxa de mortalidade dos municípios com menos de 5 000 habitantes (referência) pertencentes à região Sudeste (referência).

Devido ao período e à unidade de análise utilizada (municípios), fatores como idade na menarca e na menopausa, tipo de alimentação e percentual de fumantes não puderam ser incluídos nos modelos. Optou-se por não utilizar a cor da pele devido ao elevado percentual de dados ausentes e aos frequentes erros de classificação associados a essa variável (28). Ademais, como o DATASUS (22) passou a divulgar o número de mamógrafos por município apenas em 1999, essa variável também foi desconsiderada.

A correlação excessiva entre as variáveis explicativas (equação 3) poderia gerar multicolinearidade, comprometendo as estimações. Contudo, o teste de fator de inflação da variância (*vector inflation factor*, VIF), que mede quanto cada variável explicativa “k” está associada às demais, não indicou a presença desse problema. Formalmente, tem-se:

$$VIF=1/(1–R_{k}^{2})$$,

onde Rk2 representa o R2 tradicional oriundo da estimação da variável “k” contra as demais explicativas (29).

### Análise dos dados

Os dados foram agrupados em um painel balanceado, onde uma mesma unidade de corte transversal (neste caso, os municípios brasileiros) foi analisada ao longo do tempo, isto é, 1990, 2000 e 2010. Esta opção permitiu considerar mais observações (melhorando as propriedades assintóticas dos estimadores-estatísticas *t* e F mais robustas), controlar efeitos não observados (“c”) constantes no tempo (por exemplo, cultura, clima e relevo) e realizar análises dinâmicas (por exemplo, análise de tendência) (30).

No contexto de dados de painel, geralmente estima-se um modelo do tipo *pooledordinary least squares* (POLS) usando o teste de Breusch-Pagan para verificar se existe algum c_i_ afetando os resultados (31). Nesse caso, a hipótese nula é:

$$H_{0}:(\sigma \frac{2}{c})=0$$,

onde

$$\sigma \frac{2}{c}$$

representa a variância do efeito não observado “c”. Caso a hipótese nula prevaleça, o modelo POLS é o mais indicado. Caso a hipótese nula não se confirme, estimam-se modelos de efeitos fixos e aleatórios, usando o teste de Hausman (32) para definir o mais adequado. Esse teste verifica se as variáveis explicativas (*X*_it_) estão correlacionadas com ci, sendo *H*_0_: *E*[(*c*_i_ | *X*_it_) = 0]. Se *H*_0_for verdadeiro, ambos os modelos serão consistentes, porém o modelo aleatório será mais eficiente. Do contrário, o teste de Breusch-Pagan se torna desnecessário e somente o modelo de efeitos fixos será consistente (31).

É importante notar que somente o estimador de efeitos fixos é capaz de eliminar o viés causado por c_i_. Para tanto, utilizam-se os desvios de cada variável *x*_t_ em relação a sua media

$$\bar{x}$$,

ou seja:

$${\ddot{x}}_{t}=x_{t}-\bar{x}$$

Exemplo: considere que “Sul” seja utilizada como *dummy* para refletir a cultura dessa região. Como um município “i” da região não mudará sua localização nos anos analisados, a *dummy* será igual a 1 em 1990, 2000 e 2010, e também terá média igual a 1. Logo, a *dummy* (e qualquer outro c_i_) acaba eliminada do modelo, pois:

$${\ddot{x}}_{t}=(x_{t}-\bar{x})=(1–1)=0$$.

A variável utilizada para captar a tendência associada à mortalidade por câncer de mama foi função exclusiva do período considerado (tendência determinística), ou seja: *MCM*_*it*_ = *Tend*_*t*_ + *ε*_*t*_, onde MCM_it_ é a mortalidade de cada município “i” no período “t”; Tendt representa a tendência, com valores iguais a 1 (em 1990), 2 (em 2000) e 3 (em 2010); e εt é um erro aleatório bem comportado. Assim, foi possível avaliar a evolução da mortalidade por câncer de mama (MCM), em valores absolutos, para cada período analisado (29).

A inclusão/exclusão de variáveis se baseou no critério de informação de Akaike (AIC) e no critério de informação Bayesiano (BIC). Ambos se valem do valor da função de máxima verossimilhança como medida de ajustamento e penalizam o excesso de variáveis incluídas. Quanto menor o valor de AIC e BIC, melhor o ajustamento (29). A estratégia empírica consistiu em incluir “grupos” de variáveis (*dummies* de região e porte municipal, variável de tendência e variáveis contínuas, como renda, longevidade, educação, industrialização, fecundidade, número de mulheres jovens grávidas,população urbana/rural, gasto público em saúde, número de profissionais e estabelecimentos de saúde), a fim de verificar como a inclusão de um novo grupo afetaria os resultados previamente obtidos.

O *software* STATA foi usado para estimativas e resultados estatísticos apresentados nesta pesquisa.

## RESULTADOS

A análise descritiva das variáveis revelou que a taxa de MCM dos municípios brasileiros, padronizada por faixa etária e corrigida por causas mal definidas, foi, em média, 9,66/100 000 no período analisado. No mesmo período, verificou-se aumento do IDHR, IDHL e IDHE. Já a produção industrial *per capita* municipal foi equivalente a 1 254,14 reais (685,54 dólares) (tabela 1).

A taxa de fecundidade diminuiu de 3,20, em 1990, para 1,99 em 2010. A média do período foi de 2,59 filhos por mulher. Já o percentual de mulheres jovens com filhos cresceu. Em média, aproximadamente 2,8% das mulheres com menos de 18 anos possuíam filhos. O coeficiente (menor que 1) associado à razão população rural/população urbana indicou que a maior parte das pessoas residia em áreas urbanas.

No ano 2000, o gasto público em saúde avaliado em reais foi equivalente a R$ 101,30 *per capita* (US$ 55,37). O número de profissionais de saúde foi de 337,39/100 000 habitantes. Por sua vez, o número de estabelecimentos de saúde foi de 45/100 000 habitantes. Todas as três variáveis aumentaram no período (tabela 1).

Como mostra a tabela 1, 39% dos 2 311 municípios considerados pertencem ao Sudeste e aproximadamente 29% tinham entre 20 e 50 000 habitantes. Os valores da variável de tendência são iguais a 1 (em 1990), 2 (em 2000) e 3 (em 2010). Para a tendência associada às regiões, os valores mencionados são atribuídos apenas aos municípios da região analisada (para os demais, atribui-se o valor zero).

Os resultados da tabela 2 indicam que houve uma tendência de crescimento da mortalidade por câncer de mama no Brasil (coeficientes positivos). Verificou-se, ainda, uma diferença regional significativa associada à mortalidade. Segundo o modelo “a”, a mortalidade foi maior na região Sul (mais rica) e menor na região Norte (mais pobre). Como a região Sudeste foi a referência, sua taxa é igual à constante (9,94) mais a tendência (0,22). Logo, a taxa de mortalidade desta região foi de 10,16 (9,94 + 0,22) em 1990, 10,38 (10,16 + 0,22) em 2000 e 10,60 (10,38 + 0,22) em 2010; a média foi igual a 10,38. Já a da região Sul foi de 11,26 (9,94 + 0,22 + 1,10) (sendo 1,10 o valor arredondado do coeficiente associado a *dummy* da região Sul, de 1,098, como mostra a tabela 2) em 1990, 11,48 (11,26 + 0,22) em 2000 e 11,70 (11,48 + 0,22) em 2010, com média de 11,48. As regiões Norte, Nordeste e Centro-Oeste apresentaram as menores taxas.

**TABELA 1.** Análise descritiva das variáveis associadas ao câncer de mama em municípios brasileiros, 1990 a 2010

Variável^a^	Média	Desvio padrão	Mínimo	Máximo
Corte transversal: 1990 (observações: 2 311)				
MCM	9,53	5,88	0,47	50,74
IDHR	0,57	0,09	0,32	0,80
IDHL	0,68	0,07	0,44	0,80
IDHE	0,23	0,09	0,02	0,56
Industrialização^b, c^	874,06	2 356,17	0,01	74 216,37
Fecundidade	3,20	0,90	1,76	8,29
Mulheres jovens com filhos	2,31	1,45	0,00	9,73
População rural/população urbana	0,92	1,55	0,00	38,63
GPS^c, d^	25,02	23,37	0,00	252,15
NPS	172,43	176,19	0,00	6 434,56
NES	33,79	25,14	0,00	215,78
Corte transversal: 2000				
MCM	9,47	5,45	0,43	47,32
IDHR	0,62	0,08	0,37	0,86
IDHL	0,75	0,06	0,54	0,87
IDHE	0,42	0,11	0,12	0,74
Industrialização^b, c^	1 132,28	2 142,78	50,23	40 912,96
Fecundidade	2,58	0,52	1,56	6,31
Mulheres jovens com filhos	3,36	1,74	0,00	16,04
População rural/população urbana	0,63	1,51	0,00	63,77
GPS^c, d^	78,91	108,24	0,00	4 702,09
NPS	260,16	307,12	0,00	8 351,03
NES	39,00	23,38	0,00	214,82
Corte transversal:2010				
MCM	9,98	4,53	0,84	43,90
IDHR	0,68	0,07	0,50	0,89
IDHL	0,82	0,04	0,68	0,89
IDHE	0,60	0,08	0,33	0,81
Industrialização^b, c^	1 756,07	3 765,65	116,91	70 961,45
Fecundidade	1,99	0,37	1,21	4,89
Mulheres jovens com filhos	2,74	1,49	0,00	12,24
População rural/população urbana	0,46	0,57	0,00	5,36
GPS^c, d^	199,97	100,10	0,00	1 268,94
NPS	579,59	327,71	55,05	3 024,09
NES	62,78	27,50	0,00	303,48
Painel: 1990 a 2010 (observações: 2 311 x 3 = 6 933)				
MCM	9,66	5,32	0,43	50,74
IDHR	0,62	0,09	0,32	0,89
IDHL	0,75	0,08	0,44	0,89
IDHE	0,42	0,18	0,02	0,81
Industrialização^b, c^	1 254,14	2 870,97	0,01	74 216,37
Fecundidade	2,59	0,81	1,21	8,29
Mulheres jovens com filhos	2,80	1,62	0,00	16,04
População rural/população urbana	0,67	1,31	0,00	63,77
GPS^c, d^	101,30	113,04	0,00	4 702,09
NPS	337,39	328,91	0,00	8 351,03
NES	45,19	28,35	0,00	303,48
*Dummies* geográficas^e, f^				
Sudeste	0,39	NA	0	1
Sul	0,26	NA	0	1
Centro-Oeste	0,07	NA	0	1
Norte	0,03	NA	0	1
Nordeste	0,25	NA	0	1v
Total	1,00	
*Dummies* de porte municipal^e, f^				
Até 5 000	0,06	NA	0	1
5 a 10 000	0,15	NA	0	1
10 a 20 000	0,26	NA	0	1
50 a 100 000	0,12	NA	0	1
100 a 500 000	0,10	NA	0	1
500 000 a 1 000 000	0,01	NA	0	1
> 1 000 000	0,01	NA	0	1
Total	1,00	
Tendência^f^				
Brasil	NA	NA	1	3
Sul	NA	NA	0	3
Centro-Oeste	NA	NA	0	3
Norte	NA	NA	0	3
Nordeste	NA	NA	0	3
Sudeste	NA	NA	0	3

***Fonte:*** DATASUS, IPEADATA e PNUD.

a GPS = gasto público *per capita* em saúde; IDHE = índice de desenvolvimento humano referente a educação; IDHL = índice de desenvolvimento humano referente a longevidade; IDHR = índice de desenvolvimento humano referente a renda; MCM = taxa de mortalidade por câncer de mama/100 000 habitantes; NES = número de estabelecimentos de saúde; NPS = número de profissionais de saúde com ensino superior.

b O valor de produção industrial *per capita* (em dólares para o ano de 2000) foi: US$ 477,78 (1990); US$ 618,93 (2000); US$ 959,91 (2010). Média: US$ 685,54.

C A conversão de reais em dólares americanos baseou-se na taxa de câmbio comercial (valor de venda), referente ao ano de 2000, disponibilizada pelo Banco Central do Brasil (cotação: R$/US$ = 1,83).

d O valor de gasto público *per capita* (em dólares para o ano de 2000) foi: US$ 13,68 (1990); US$ 43,13 (2000); US$ 109,31 (2010). Média: US$ 55,37.

e As médias das *dummies* geográficas e de porte municipal refletem a proporção de municípios pertencentes a cada categoria.

f NA = não se aplica.

**TABELA 2.** Modelos estimados para os municípios brasileiros contendo variáveis discretas associadas ao câncer de mama, 1990 a 2010

Variável	Modelo^a^				
	(a)	(b)	(c)	(d)
Região			
Sul	1,098^b^	0,304	1,117^b^	0,322
Centro-Oeste	-2,183^b^	-4,250^b^	-1,917^b^	-3,984^b^
Norte	-4,264^b^	-6,311^b^	-4,420^b^	-6,467^b^
Nordeste	-2,958^b^	-5,493^b^	-2,718^b^	-5,252^b^
Sudeste	Omitido	Omitido	Omitido	Omitido
Tendência			
Brasil	0,221^c^	0,221^c^	
Sul	0,096	0,096
Centro-Oeste	0,732^c^	0,732^c^
Norte	0,722	0,722
Nordeste	0,966^b^	0,966^b^
Sudeste^e^	-0,302^d^	-0,302^c^
Porte municipal (população)			
< 5 000	Omitido	Omitido
5 a 10 000	-2,802^b^	-2,802^b^
10 a 20 000	-3,622^b^	-3,622^b^
20 a 50 000	-3,769^b^	-3,769^b^
50 a 100 000	-3,059^b^	-3,059^b^
100 a 500 000	-1,209^b^	-1,209^b^
500 a 1 000 000	1,215	1,215
> 1 000 000	3,222^b^	3,222^b^
Constante	9,941^b^	10,986^b^	12,776^b^	13,821^b^
R^2^	0,10	0,11	0,16	0,16
AIC^f^	42 132,92	42 090,85	41 708,71	41 663,36
BIC^f^	42 173,98	42 159,29	41 797,69	41 779,71

a Coeficientes de mortalidade por 100 000 habitantes. Modelos estimados via *pooled ordinary least squares* (POLS). Modelos “a”: inclui *dummies* de região + tendência e variável de tendência geral, válida para o Brasil (média dos municípios brasileiros). Modelos “b”: inclui *dummies* de região + tendência + variáveis de tendência regional (uma para cada região). Modelo “c”: inclui *dummies* de região + variável de tendência geral + porte municipal. Modelo “d”: inclui *dummies* de região + variável de tendência regional + porte municipal.

b *P* < 0,001 (teste t de Student).

c *P* < 0,01 (teste t de Student).

d *P* < 0,05 (teste t de Student).

e Os municípios do Sudeste e aqueles com menos de 5 000 habitantes são referência e, portanto, foram omitidos das estimações.

f AIC = critério de informação de Akaike; BIC = critério de informação Bayesiano.

**TABELA 3.** Modelos estimados para os municípios brasileiros contendo variáveis contínuas associadas ao câncer de mama, 1990 a 2010

Variável^b^	Modelo^a^			
	(a) POLS^c^	(b) RE^d^	(c) FE^e^
IDHR	21,039^f^	20,356^f^	2,365
IDHL	2,540	3,218	6,238^g^
IDHE	-5,352^f^	-5,332^f^	-0,569
Industrialização	0,028	0,025	-0,015
Fecundidade	-0,468^f^	-0,382^h^	-0,018
Mulheres jovens grávidas	-0,196^f^	-0,172^f^	-0,032
População rural/urbana	-0,010	-0,004	0,022
GPS	-0,086	-0,140^g^	-0,174^g^
NPS	0,049^e^	0,033	-0,029
NES	0,115	0,014	0,051
Constante	-1,524	-1,755	4,148
R^2^	0,11	0,11	0,08
AIC^i^	42 032,87	-	37 509,62
BIC^i^	42 108,16	-	37 584,90

a Coeficientes de mortalidade por 100 000 habitantes. Teste de Hausman (1978): (c) *versus* (b):χ^2^ = 523,41; probabilidade >χ^2^ = 0,000.

b GPS = gasto público *per capita* em saúde; IDHE = índice de desenvolvimento humano referente a educação; IDHL = índice de desenvolvimento humano referente a longevidade; IDHR = índice de desenvolvimento humano referente a renda; NES = número de estabelecimentos de saúde; NPS = número de profissionais de saúde com ensino superior.

c POLS = *pooled ordinary least squares*.

d RE = *random effects*.

e FE = *fixed effects*.

f *P* < 0,001 (t de Student).

g *P* < 0,05 (t de Student).

h *P* < 0,01 (t de Student).

i AIC = critério de informação de Akaike; BIC = critério de informação Bayesiano.

Segundo o modelo “b”, as regiões Nordeste (0,97), Centro-Oeste (0,73) e Norte (0,72) foram as grandes responsáveis pela tendência de crescimento da mortalidade (tabela 2). Verificou-se ainda uma tendência negativa na região Sudeste (-0,30) e uma estabilidade no Sul (onde o valor não foi significativo).

Segundo as *dummies* de porte municipal, modelo “c”, as menores taxas de mortalidade são encontradas em municípios com 20 a 50 000 habitantes. Já as maiores ocorreriam naqueles com mais de 1 milhão de habitantes (9, 22). Taxas elevadas também foram verificadas nos municípios com população entre 500 000 e 1 milhão e naqueles onde a população não chegava a 5 000.

A inclusão conjunta das variáveis de porte municipal, região e tendência, modelos “c” e “d”, afetou a magnitude, mas não a ordenação dos resultados. Portanto, presume-se que as análises anteriores permaneceram válidas (tabela 2).

O teste de Hausman (23) indicou que o modelo de efeitos fixos (tabela 3) foi preferível aos demais. Segundo esse modelo, a mortalidade por câncer de mama apresentou uma associação positiva/significativa com a longevidade e negativa/significativa com o nível de gastos públicos em saúde.

Diferentemente dos resultados descritos na tabela 2, os modelos da tabela 4 indicaram uma tendência negativa/significativa associada à mortalidade por câncer de mama (modelos “a” e “b”). Os modelos “c” e “d” revelaram que essa tendência negativa ocorreu em todas as regiões, sendo mais intensa no Sudeste (a cada decênio, a taxa diminuiria entre -2,66 e -2,67/100 000 mulheres) e no Sul (cerca de -2,29 a -2,30/100 000 mulheres).

A inclusão das variáveis explicativas contínuas não afetou a ordenação das *dummies* de região e porte municipal, mostrando que as análises anteriores permanecem válidas (tabela 4). A redução dos AIC e BIC apresentada na tabela 4, em relação aos resultados das tabelas 2 e 3, indicou uma melhora na especificação dos modelos. Apesar disso, o teste de Hausman (32) revelou que não foi possível eliminar completamente o problema causado pelos efeitos não observados. Logo, os resultados dos modelos de efeitos fixos foram os mais confiáveis.

## DISCUSSÃO

Os resultados desta pesquisa indicam que a taxa de mortalidade por câncer de mama, padronizada por faixa etária e corrigida por causas mal definidas, cresceu no Brasil entre 1990 e 2010. Essa tendência difere da verificada em países desenvolvidos, onde a mortalidade é declinante (3, 14). É possível que fatores externos, como o rescimento da renda e da longevidade e a diminuição da taxa de fecundidade, tenham contribuído para esse crescimento.

As estimações indicaram que a mortalidade apresenta associação positiva/significativa com a longevidade e negativa/significativa com o nível de gastos públicos em saúde. O resultado associado ao gasto público é coerente com o relatório da Organização Mundial da Saúde (OMS) (33), segundo o qual a redução de doenças não transmissíveis, como o câncer de mama, ainda requer um aumento significativo nos gastos com saúde em países em desenvolvimento. Quanto à longevidade, o achado é semelhante aos descritos na literatura (1, 14, 16).

As *dummies* de região indicaram que a mortalidade é maior no Sul e Sudeste e menor no Norte, Nordeste e Centro-Oeste, respectivamente. Em parte, essa desigualdade se deve às peculiaridades regionais e já havia sido identificada anteriormente (13). Na realidade, os níveis elevados de renda e longevidade e a baixa taxa de fecundidade das regiões Sul e Sudeste, em relação às demais, já explicariam parte dessa diferença.

A consideração de algumas características socioeconômicas municipais mostrou que os coeficientes associados às *dummies* regionais se aproximaram dezero. Portanto, as desigualdades socioeconômicas podem ter sido responsáveis por parte da diferença regional associada à mortalidade por câncer de mama. De fato, Sul e Sudeste permaneceram com taxas maiores que as demais regiões.

**TABELA 4.** Modelos estimados para os municípios brasileiros contendo variáveis discretas e contínuas associadas ao câncer de mama, 1990 a 2010

Variáveis contínuasa	Tendência geral		Tendência regional		Efeitos fixos
	(a) POLS^b^	(b) RE^c^	(c) POLS^b^	(d) RE^c^	(e) FE^d^
IDHR	9,063^e^	8,728^e^	9,267^e^	8,856^e^	2,365
IDHL	8,098^e^	8,698^e^	6,114^f^	6,303^f^	6,238^g^
IDHE	5,865^e^	6,137^e^	6,754^e^	7,193^e^	-0,569
Industrialização	0,018	0,015	0,021	0,018	-0,015
Fecundidade	-0,123	-0,115	0,061	0,095	-0,018
Mulheres jovens com filhos	0,040	0,033	0,019	0,012	-0,032
População rural/população urbana	-0,125^f^	-0,108^g^	-0,120^g^	-0,102^g^	0,022
GPS	0,061	0,029	0,094	0,065	-0,174^g^
NPS	0,074^f^	0,060^g^	0,088^e^	0,075^f^	-0,029
NES	-0,542^g^	-0,415	-0,603^g^	-0,487	0,051
Região				
Sul	0,969^e^	0,935^e^	0,253	0,218	Omitido
Centro-Oeste	-1,418^e^	-1,420^e^	-3,392^e^	-3,363^e^	Omitido
Norte	-2,505^e^	-2,532^e^	-4,697^e^	-4,754^e^	Omitido
Nordeste	0,123	0,111	-2,207^e^	-2,264^e^	Omitido
Sudeste	Omitido	Omitido	Omitido	Omitido	Omitido
Porte municipal (população)				
< 5 000	Omitido	Omitido	Omitido	Omitido	Omitido
5 a 10 000	-2,778^e^	-2,765^e^	-2,783^e^	-2,772^e^	Omitido
10 a 20 000	-3,626^e^	-3,600^e^	-3,641^e^	-3,619^e^	Omitido
20 a 50 000	-4,283^e^	-4,241^e^	-4,309^e^	-4,270^e^	Omitido
50 a 100 000	-4,273^e^	-4,216^e^	-4,300^e^	-4,243^e^	Omitido
100 a 500 000	-3,062^e^	-2,991^e^	-3,105^e^	-3,034^e^	Omitido
500 a 1000 000	-1,884^f^	-1,771^g^	-1,944^f^	-1,826^g^	Omitido
> 1 000 000	-0,553	-0,419	-0,628	-0,487	Omitido
Tendência				
Brasil	-2,222^e^	-2,246^e^	Omitido
Sul	-2,289^e^	-2,296^e^	Omitido
Centro-Oeste	-1,663^e^	-1,690^e^	Omitido
Norte	-1,613^e^	-1,623^e^	Omitido
Nordeste	-1,556^e^	-1,563^e^	Omitido
Sudeste^h^	-2,664^e^	-2,674^e^	Omitido
Constante	3,469^g^	3,152^g^	4,902^e^	4,784^f^	4,148
R^2^	0,21	0,21	0,21	0,21	0,08
AIC^i^	41299,18	-	41268,08	-	37509,62
BIC^i^	41456,59	-	41452,86	-	37584,90

a Coeficientes de mortalidade por 100 000 habitantes. GPS = gasto público *per capita* em saúde; IDHE = índice de desenvolvimento humano referente a educação; IDHL = índice de desenvolvimento humano referente a longevidade; IDHR = índice de desenvolvimento humano referente a renda; MCM = taxa de mortalidade por câncer de mama/100 000 habitantes; NES = número de estabelecimentos de saúde; NPS = número de profissionais de saúde com ensino superior.

b POLS = *pooled ordinary least squares*.

c RE = *random effects*.

d FE = *fixed effects*. O estimador FE elimina automaticamente as variáveis que não apresentam variação entre os anos analisados (omitido).

e *P* < 0,001 (teste t de Student).

f *P* < 0,01 (teste t de Student).

g *P* < 0,05 (teste t de Student).

h Os municípios do Sudeste e aqueles com menos de 5 000 habitantes são referência e, portanto, foram omitidos das estimações.

i AIC e BIC são os critérios de informação de Akaike e Bayesiano, respectivamente.

A análise de *dummies* de porte municipal mostrou as menores taxas de mortalidade nos municípios com 20 a 50 000 habitantes. Já as maiores ocorreram tanto nos municípios com mais de 500 000 habitantes quanto naqueles onde a população não chega a 5 000. No caso dos municípios pequenos, é possível que a falta de infraestrutura dificulte a detecção e o tratamento da doença. Por sua vez, nas localidades de grande porte, a elevada mortalidade poderia ser um reflexo do deslocamento de mulheres com câncer de mama para os grandes centros embusca de melhores tratamentos, além da urbanização e possíveis mudanças nos padrões reprodutivos. Gebrim e Quadros já haviam detectado esse gargalo nas grandes cidades do Brasil (20).

Inicialmente, a variável de tendência indicou um aumento significativo na taxa de mortalidade entre 1990 e 2010 no Brasil (tabela 2). Esse crescimento foi maior no Nordeste, Centro-Oeste e Norte, estável no Sul e negativo no Sudeste, o que é condizente com relatos anteriores (34, 35). É possível que o crescimento da renda e da longevidade e a redução na taxa de fecundidade verificados no Brasil no período considerado tenham impulsionado esse aumento na mortalidade. Quanto ao Sudeste, por ser a região mais rica, acredita-se que seus avanços na prevenção, detecção e tratamento do câncer de mama estejam permitindo uma redução na mortalidade, assim como em países mais desenvolvidos.

É interessante notar que, após considerar algumas variáveis socioeconômicas (isto é, renda, longevidade, fecundidade), a tendência associada à taxa de mortalidade se tornou negativa/significativa em todas as regiões e foi mais intensa no Sudeste e Sul e menor no Nordeste (tabela 4). A explicação para a inversão da tendência requer certa atenção. De modo geral, os resultados da tabela 2, sem considerar as variáveis contínuas (renda, longevidade, fecundidade), indicaram um crescimento da mortalidade (tendência positiva). Porém, após inclusão das variáveis contínuas, a tendência se tornou negativa, mostrando o que ocorreria com a mortalidade se tais fatores não tivessem variado no período. Entretanto, a renda e a longevidade cresceram entre 1990 e 2010, enquanto a fecundidade diminuiu.Logo, como a renda, a longevidade, e a fecundidade apresentaram associações positiva, positiva e negativa em relação à mortalidade, respectivamente, é possível inferir que a variação desses fatores contribuiu, pelo menos em parte, para o crescimento verificado na mortalidade. Em outras palavras, caso fatores externos como renda, expectativa de vida e taxa de fecundidade, entre outros, não tivessem se alterado no período, as taxas de mortalidade poderiam ter sido reduzidas nas regiões brasileiras.

Apesar dos gastos públicos em saúde terem aumentado no período, ainda há necessidade de investimentos nessa área, principalmente em localidades de pequeno e grande porte (com menos de 5 000 e mais de 500 000habitantes, respectivamente), onde a mortalidade se mostrou acima da média.

Cabe destacar que o uso de dados secundários agregados e a impossibilidade de inclusão de alguns fatores associados à mortalidade por câncer de mama constituem uma limitação deste trabalho. Além disso, a análise da mortalidade ao longo de 20 anos envolve outros aspectos de difícil incorporação nos modelos (por exemplo, avanços/alterações nos registros de óbito, nos diagnósticos e nos tratamentos do câncer de mama). Apesar disso, este trabalho permitiu identificar alguns fatores socioeconômicos que, possivelmente, contribuíram para o crescimento da mortalidade por câncer de mama no Brasil (por exemplo, crescimento da renda e da longevidade e redução da fecundidade). Foi ainda possível distinguir algumas características dos municípios com elevada mortalidade (porte pequeno/grande e pertencer às regiões Sul e Sudeste) e propor medidas para reduzir a mortalidade média noLogo, como a renda, a longevidade, e a fecundidade apresentaram associações positiva, positiva e negativa em relação à mortalidade, respectivamente, é possível inferir que a variação desses fatores contribuiu, pelo menos em parte, para o crescimento verificado na mortalidade. Em outras palavras, caso fatores externos como renda, expectativa de vida e taxa de fecundidade, entre outros, não tivessem se alterado no período, as taxas de mortalidade poderiam ter sido reduzidas nas regiões brasileiras.

Cabe destacar que o uso de dados secundários agregados e a impossibilidade de inclusão de alguns fatores associados à mortalidade por câncer de mama constituem uma limitação deste trabalho. Além disso, a análise da mortalidade ao longo de 20 anos envolve outros aspectos de difícil incorporação nos modelos (por exemplo, avanços/alterações nos registros de óbito, nos diagnósticos e nos tratamentos do câncer de mama). Apesar disso, este trabalho permitiu identificar alguns fatores socioeconômicos que, possivelmente, contribuíram para o crescimento da mortalidade por câncer de mama no Brasil (por exemplo, crescimento da renda e da longevidade e redução da fecundidade). Foi ainda possível distinguir algumas características dos municípios com elevada mortalidade (porte pequeno/grande e pertencer às regiões Sul e Sudeste) e propor medidas para reduzir a mortalidade média nopaís (por exemplo, expandir os gastos públicos em saúde). Obviamente, deve-se considerar que boa parte dos municípios com baixas taxas de mortalidade são, na realidade, menos favorecidos (pobres) e, portanto, também precisarão de mais investimentos e políticas voltadas ao tratamento e detecção desta neoplasia à medida que suas rendas e taxas de longevidade aumentarem.

Como é possível que outras nações emergentes enfrentem transições socioeconômicas e demográficas semelhantes às vivenciadas pelo Brasil, é provável que também experimentem uma elevação significativa da mortalidade por câncer de mama. Portanto, é necessário que tais países estejam preparados. Com base nos resultados descritos aqui, é possível inferir que a mortalidade nesses países tenderá a se concentrar, primeiramente, nas grandes/pequenas cidades de suas regiões mais ricas.

### Agradecimentos.

Os autores agradecem à Coordenação de Aperfeiçoamento de Pessoal de Nível Superior (CAPES) pela bolsa de estudos (mestrado) concedida a MSAC durante a realização desta pesquisa. MTBT é bolsista de produtividade do Conselho Nacional de Desenvolvimento Científico e Tecnológico (CNPq).

### Declaração.

As opiniões expressas no manuscrito são de responsabilidade exclusiva dos autores e não refletem necessariamente a opinião ou política da RPSP/PAJPH ou da Organização Pan-americana de Saúde (OPAS).
